# Prevalence of obesity, diabetes mellitus, hypertension and associated risk factors in a mining workforce, Democratic Republic of Congo

**DOI:** 10.11604/pamj.2017.28.282.14361

**Published:** 2017-11-30

**Authors:** Paul Makan Mawaw, Thierry Yav, Olivier Mukuku, Olivier Lukanka, Patrick Mumba Kazadi, Daniel Tambwe, Jules Omba, Jean-Baptiste Kakoma, Michael John Bangs, Oscar Numbi Luboya

**Affiliations:** 1 Department of Public Health, University of Lubumbashi, Democratic Republic of Congo; 2 Department of Research, High School for Medical Technics of Lubumbashi, Democratic Republic of Congo; 3 Occupational Health Clinic, International SOS Tenke Fungurume Mine, Democratic Republic of Congo; 4 International SOS Clinic, Kinshasa, Democratic Republic of Congo; 5 Anglo-Gold Ashanti, Mongbwalu Clinic, Democratic Republic of Congo; 6 Department of Gynecology and Obstetrics, University of Lubumbashi, Democratic Republic of Congo; 7 Freeport/International SOS Public Health and Malaria Control, Kuala Kencana, Papua, Indonesia

**Keywords:** Obesity, diabetes mellitus, hypertension, risk factors, workforce, mining, Democratic Republic of Congo

## Abstract

**Introduction:**

The burden of non-communicable diseases (NCDs) is increasing in low and middle-income countries (LMIC). According to the World Health Organization (WHO) the largest increase occurs in Africa. Obesity, diabetes mellitus and hypertension (ODH) are major risk factors for cardiovascular diseases, causing nearly 18 million deaths worldwide. Various risks associated with mining as an occupational activity are implicated in NCDs' occurrence. This study describes the baseline prevalence of ODH and associated risk factors in the workforce of Tenke Fungurume Mining (TFM), in southern Democratic Republic of Congo.

**Methods:**

A cross-sectional study was conducted on a sample of 2,749 employees' and contractor's occupational health examination files for 2010. Socio-demographic, occupational, medical, anthropometric and behavioral characteristics were collected and assessed. Disease status regards ODH was based on WHO criteria. A multivariate logistic regression model was used.

**Results:**

Overall prevalence of ODH was 4.5%, 11.7%, and 18.2% respectively. Proportions of pre-ODH individuals were 19.7%, 16.5%, and 47.8% respectively. Prevalence of ODH increased with age, professional grade, nature of work, gender and reported alcohol use. Smoking 10 or more cigarettes per day increased risk of diabetes and hypertension, while decreasing obesity.

**Conclusion:**

Rates of ODH and associated risk factors are higher in the TFM workforce, than in the general DRC population. This is likely reflective of other mining sites in the country and region. It is evident that ODH are associated with various socio-demographic, occupational, anthropometric, biomedical and behavioral risk factors. A NCD prevention program and close monitoring of disease and risk factors trends are needed in this population.

## Introduction

Non-communicable diseases (NCDs) currently result in more premature deaths than all other causes combined. Together they kill nearly 40 million people each year, accounting for 70% of all global deaths [1, 2]. According to World Health Organization (WHO), if unabated, the number of deaths will reach 52 million annually by 2030 [3]. Cardiovascular diseases (CVD), cancers, chronic respiratory diseases and diabetes mellitus (Type 2) are the four main NCDs attributable to common risk factors [4, 5]. They currently cause an estimated 82% of all NCD-related deaths namely: CVD (17.7 million deaths; 46.2%), cancers (8.8 million; 21.7%), chronic obstructive pulmonary disease (3.9 million; 10.7%) and diabetes (1.6 million; 4%) [1, 6]. In 2015, nearly 80% of NCD related deaths (30.7 million) occurred in low-and middle-income countries (LMICs), with approximately 48% occurring in adults and less than 70 years of age [2]. Because of relatively higher birth rates compared to developed nations and a rapid shift in life-style, the burden of NCDs is increasing rapidly in many LMICs. The WHO projects a 17% increase in global NCD mortality over the coming decades, the largest of which (24%) will occur in Africa [7]. Obesity, diabetes mellitus and hypertension (ODH) are major risk factors for CVD, the cause of nearly 18 million deaths annually worldwide [8]. Overweight and obesity, defined as a body mass index (BMI) of ≥ 25 kg/ m² and ≥ 30 kg/m^2^, respectively, were linked to 3.4 million global deaths and 93.6 million Disability Adjusted Life Years (DALYs) in 2010 [4]. Between 2010 and 2014, the prevalence of overweight adults aged 18 and over in the Democratic Republic of Congo (DRC) had increased from 18.8% to 20.6%; while obesity rates rose from 3.7 to 4.4% [6]. By 2030, the number of diabetics is expected to reach 366 million globally. This increase will be more marked in developing countries, where the number of diabetics will rise from 84 million to 228 million [9]. Currently, the prevalence of diabetes in African countries ranges between 2 and 9% [10]. In the DRC, it has increased steadily from 5.7% to 6.1% between 2010 and 2014 [6]. It is estimated that more than 1 billion people in the world suffered from hypertension in 2000, and this number is expected to rise to nearly 1.6 billion by 2025 [11]. Hypertension is the leading CVD risk factor in sub-Saharan Africa, where more than 20 million people were already affected before 1995 [12]. According to WHO, hypertension was identified as the greatest NCD problem in the DRC in 2014; with a prevalence estimated at 24.8% and one of the highest in Africa [6].

A baseline health survey (BHS) conducted in 2008 in the Tenke Fungurume Mining (TFM) concession located in southern DRC found 12% of respondents acknowledging they had hypertension [13]. Additionally, the same BHS found that prevalence of overweight and obesity in both sexes was 12.7% and 4.7%, respectively. Mining represents one of the most hazardous occupational activities in the world, both in terms of potential acute injuries and deaths, but also long-term health impacting exposures, resulting in various cancers and chronic respiratory diseases. Environmental exposure risks associated with mining have been implicated in the occurrence or exacerbation of NCDs. For example, occupational exposures to excessive (high decibel) noise and body vibration have been linked with increased risk for cardiovascular morbidity and mortality [14, 15]. Increased levels of work-related stress, isolated living conditions, catered food service, night shift work and other factors associated with the mining industry contribute to the magnification of important risk factors resulting in disease [16, 17]. In 2011, the United Nations General Assembly formally acknowledged that NCDs represent a significant challenge for development in the 21^st^ century and called for direct involvement of the private sector for strengthening its contribution to prevention and control [18]. The TFM is currently the largest mining operation in the DRC. The TFM workforce is made up of a large portion of young to middle aged male adults and generally representative of a cross section of DRC middle income society. The vast majority of workers have at least a secondary school education level. Despite the importance of mining to the national economy and welfare, until now, studies on prevalence and impact of NCDs and their risk factors in the mining industry have been lacking in the DRC. The aim of this initial study is to describe the burden of ODH and associated risk factors in the TFM workforce, using 2010 occupational health records. Data and analysis will serve as “baseline” information for follow-up comparison of NCDs trends in this population to gage changes in burden of disease and impact on individual health and productivity. A better understanding of the issues and detrimental consequences to the mining sector will serve as a foundation to develop and implement integrated intervention programs to stem the alarming rise and burden of NCDs.

## Methods

**Figure 1:**
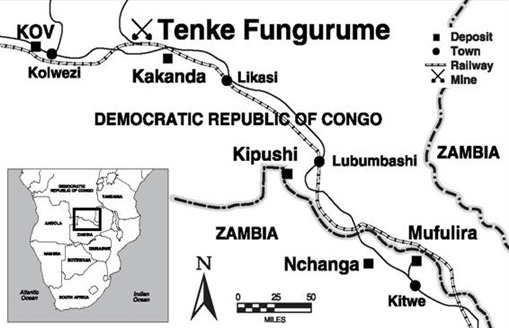
Tenke Fungurume Mining site location

**Study site:** This investigation was conducted at the TFM site, a predominately rural location, approximately 90km southeast of Kolwezi, at the altitude of 1,183m above the sea level, in Lualaba Province, DRC (Figure 1). TFM is currently the largest extractive mine operation (copper and cobalt minerals) in the DRC, with a workforce composed of primarily DRC nationals and a small percentage of expatriates. Employment recruitment began as early as 2006 during the initial project development phase. Currently, the workforce is dedicated to the project production phase that officially begun in March 2009.

**Retrospective data:** A cross-sectional study was conducted on 2,749 study subjects, all TFM employees or affiliated contractors who underwent an occupational health examination during the initial recruitment process or as post-employment follow-up between January and December 2010. In December 2016, archived medical records were reviewed and selected data gathered and transcribed electronically into an Epi Info^TM^ (CDC, Atlanta, GA, USA) database. Data on study subjects was contained in confidential Occupational Health Clinic files present on hard copies at the TFM mine site. Study data contained in the health files included:

**Socio-demographic and occupational parameters:** Gender, age, nationality, professional category and nature of work (i.e. clerical or non-clerical), occupational exposures-inhalation (dusts, vapors, smoke), chemicals, noise and vibration-and permanent residence (urban or rural).

**Anthropometric and medical parameters:** Weight, height, systolic and diastolic blood pressure, fasting blood glucose, total cholesterol, history of hypertension, diabetes, heart disease and hypercholesterolemia.

**Behavioral characteristics:** Status as current smoker and alcohol use.

**Diagnostic criteria:** BMI is universally expressed in kg/m^2^, resulting from mass in kilograms and height in meters. Overweight and obesity were defined as a BMI ≥ 25-29.9 kg/m^2^ and ≥30 kg/m^2^, respectively [6]. Diabetes was defined as the proportion of individuals with a fasting glucose level ≥ 126 mg/dl, or known diabetic with blood glucose lower than 126 mg/dl and under hypoglycemic therapy. Hypertension was defined as the proportion of individuals with a systolic blood pressure (SBP) ≥ 140 mmHg and/or a diastolic blood pressure (DBP) ≥ 90 mmHg, or a history of hypertension with normal SBP and DBP on antihypertensive therapy.

**Smoking and alcohol use behavior:** Alcohol consumption was based on reported use by study subject, entered into the Occupational Health examination form. The consumption of four or more standard units of alcohol per day was deemed the cut-off measure. One 'unit' is the equivalent 10ml (~8 g) pure ethyl alcohol and depending on the amount consumed and percentage alcohol per volume, a typical drink might contain between 1-3 units. Smoking behavior was restricted to individuals who currently smoke tobacco every day. Cigarette use was divided between those reporting having smoked on average less than 10 cigarettes and those who smoke ≥ 10 per day.

**Analysis:** Data were encoded with Epi Info^TM^ 7.1.4.0 aggregated and analyzed using Stata^®^ 14 (StataCorp LLC, College Station, TX, USA). Data analysis was performed using a combination of descriptive and inferential statistics. A t-test was performed to compare means of quantitative variables between groups based on defined parameters and characteristics and the non-parametric chi-square test (with or without Yates' correction) was used to compare proportions between defined groups. The statistical significance was set at 5% (p < 0.05).

**Ethical considerations:** Pre-employment medicals and annual follow-up medical examinations are employment requirements at TFM. Employee medical records are securely stored with controlled access at the Occupational Health Clinic at the mine site. All study parameters were initially collected by the company's qualified health care staff. Therefore, no additional biological samples or interviews were necessary for this study. All selected data that were transcribed and used in the analysis were treated with the strictest confidentiality and no personal-linked or identifiable information was disclosed outside the study team at any time. Permission to use data for research was granted by Freeport and International SOS management. Ethical review and clearance was obtained from the Medical Ethics Committee at the Faculty of Medicine, University of Lubumbashi, DRC.

## Results

**Socio-demographic and occupational characteristics of study subjects:** The reviewed medical records included 2,749 individuals: 2,574 adults males (93.6%) and 175 females (6.4%). Main socio-demographic and occupational parameters are presented in Table 1. Overall mean age of workforce was 37.8 ± 8.9 years. Females were slightly younger (36 ± 8.4 years,) than males (38 ± 9 years). The majority of study subjects (61.4%) were 40 years of age or less. The proportion of men aged 40 years and more was 39.2%, while that of women was 28%. For professional grade, 63.8% of workforce was in the lowest grade where males made up 65.1% and women 44.9% of this category. When considering the nature of work, 36.2% of subjects had a clerical job (34.9% for males and 56% for females). Only 606 (22%) of study subjects had a recorded history of potential occupational exposure risk, of which 51.5% reported some direct exposure and/or inhalation to one or more chemicals (acid, sulfur, pesticides and other chemicals, vapors and smoke), while 48.5% reported exposure to physical hazards (noises or vibrations).

**Table 1:** Socio-demographic and occupational characteristics of study subjects (2010)

Variable	Total (N=2749)		Males (n=2574)		Females (n=175)	
**Age**						
Mean (SD) [95%IC]	37.9 (8.9) [37.5- 38.2]		38.0 (9.0) [37.6 – 38.3]		36.0 (8.4) [34.8 – 37.3]	
18-29 years	493	(17.9%)	454	(17.6%)	39	(22.3%)
30-34 years	658	(23.9%)	613	(23.8%)	45	(25.7%)
35-39 years	538	(19.6%)	496	(19.3%)	42	(24.0%)
40-44 years	414	(15.1%)	393	(15.3%)	21	(12.0%)
≥45 years	646	(23.5%)	618	(24.0%)	28	(16.0%)
**Permanent residence**						
Urban	1,164	(42.3%)	1,057	(41.0%)	107	(61.1%)
Rural	1,585	(57.7%)	1,517	(59.0%)	68	(38.9%)
**Nationality**						
Congolese	2,675	(97.3%)	2,512	(97.6%)	163	(93.1%)
Expatriate	74	(2.7%)	62	(2.4%)	12	(7.9%)
**Employer**						
TFM	2,573	(93.6%)	2,438	(94.7%)	135	(77.1%)
International SOS	126	(36.6%)	94	(3.7%)	32	(18.3%)
Congo Equipment	50	(1.8%)	42	(1.6%)	8	(4.6%)
**Duration of employment (years)**						
Mean (SD)	1.3 (0.8)		1.3 (0.8)		1.2 (0.8)	
**Professional grade**						
Managers	286	(10.4%)	253	(9.8%)	33	(18.9%)
Foremen	710	(25.8%)	645	(25.1%)	65	(37.1%)
General labor/technicians	1,753	(63.8%)	1,676	(65.1%)	77	(44.0%)
**Nature of the position**						
Clerical/Administrative	996	(36.2%)	898	(34.9%)	98	(56.0%)
Non-clerical	1,753	(63.8%)	1,676	(65.1%)	77	(44.0%)
**History of occupational exposure**						
Yes	606	(22.0%)	565	(22.0%)	41	(23.4%)
No	2,143	(78.0%)	2,009	(78.0%)	134	(76.6%)
**Type of occupational exposure**	^+^(N=606)		^+^(n=565)		^+^(n=41)	
Noise	117	(19.3%)	113	(20.0%)	4	(9.8%)
Vibration	177	(29.2%)	169	(29.9%)	8	(19.5%)
Inhalation	142	(23.4%)	134	(23.7%)	8	(19.5%)
Chemicals	170	(28.1%)	149	(26.4%)	21	(51.2%)

+ Data on occupational exposure available for 606 (22%) study subjects. N/n= sample size; SD= standard deviation statistic

**Anthropometric, medical, metabolic and behavioral characteristics of study subject:** Anthropometric, medical (metabolic) and behavioral characteristics are presented in Table 2. Mean BMI for both genders was 22.9 ± 3.6 kg/m^2^. Females had a significantly greater (p < 0.0001) mean BMI (24.2 ± 5.1 kg/m^2^) than males (22.9 ± 3.5 kg/m^2^). Individuals over 40 years of age had an average BMI significantly (p < 0.0001) greater than their working counterparts under 40 years (23.3 ± 4 kg/m^2^ and 22.7 ± 3.3 kg /m^2^ respectively). The proportion of individuals with BMI < 18.5 kg/m^2^ was 6.6% and significantly (p < 0.0001) higher in males than females. Mean fasting blood glucose for both sexes was 93.9 ± 17.5 mg/dl. Females had a significantly (p < 0.0001) more elevated level 99.4 ± 22.3 mg/dl than that recorded in males 93.5 ± 17.5mg/dl. Subjects aged 40 years or greater had significantly (p < 0.0001) higher mean fasting blood glucose levels than those aged below 40 years (96.0 ± 19.5 mg/dl and 92.5 ± 16.6 mg/dl). Hypoglycemia (fasting glucose < 70.2 mg/dl) was detected in 2.2% of study subjects. The mean blood cholesterol level was 118.8 ± 36.7 mg/dl and significantly (p < 0.0001) higher in females (128.5 ± 44.3 mg /dl) than males (118.1 ± 36 mg/dl). Individuals aged 40 years and older had a higher (p < 0.0001) mean cholesterol level (131.6 mg/dl ± 39.2) than those aged below 40 years (110.6 ± 32.5 mg/dl). Blood pressure measures revealed a mean SBP of 124.0 ± 10.2 mmHg; significantly (p < 0.0001) higher in males at 124.2 ± 10.1 mmHg than in females at 120.3 ± 10.2 mmHg. The mean DBP was 79.2 ± 8.3 mmHg. There was no difference between males and females (p = 0.89). The overall prevalence of reported tobacco smoking, regardless of number of cigarettes smoked, was 18.9%. It was significantly (p < 0.0001) greater in males (19.9%) than females (4.6%). Forty-eight percent of smokers reported using more than 10 cigarettes a day. The proportion of individuals (n = 1,104) who regularly drank some form of alcohol (below or above 4 standard units) was 40.2%; being significantly (p < 0.005) higher in males (40.9%) than females (29.7%). Rate of those reporting alcohol use and consuming 4 or more units per day was 18.5%. Subjects aged less than 40 years had a significantly greater prevalence of reported alcohol use (p < 0.0001) and tobacco smoking (p < 0.05).

**Table 2:** Anthropometric, medical, metabolic and behavioral characteristics of study subjects (2010)

Variable	Total (N=2749)		Males (n=2574)		Females (n=175)	
**BMI** (kg/m^2^)						
Mean (SD) [95%CI]	22.9 (3.6) [22.8 – 23.1]		22.9 (3.5) [22.7 – 23.0]		24.2 (5.1) [23.4 – 24.9]	
Underweight (<18.5)	180	(6.6%)	170	(6.6%)	10	(5.7%)
Normal (18.5 – 24.5)	1,904	(69.3%)	1,798	(69.8%)	106	(60.5%)
Overweight (25 – 29.9)	541	(19.7%)	505	(19.6%)	36	(20.6%)
Moderate obesity (30 – 39,9)	118	(4.3%)	99	(3.9%)	19	(10.9%)
Severe obesity (≥40)	6	(0.2%)	2	(0.1%)	4	(2.3%)
**Fasting Blood glucose (mg/dl)**						
Mean (SD) [95%CI]	93.9 (17.5) [93.2 – 94.5]		93.5 (17.5) [92.8 – 94.2]		99.4 (22.3) [96.0 – 102.7]	
Hypoglycemia (<70)	62	(2.3%)	58	(2.3%)	4	(2.3%)
Normal (70,2-100)	2,075	(75.5%)	1,962	(76.2%)	113	(64.6%)
Impaired (101-125)	454	(16.5%)	420	(16.3%)	34	(19.4%)
Raised (≥126)	158	(5.7%)	134	(5.2%)	24	(13.7%)
**Serum total cholesterol (mg/dl)**						
Mean (SD) [95%CI]	118.8 (36.7) [117.4 – 120.2]		118.1 (36.0) [116.7 – 119.5]		128.5 (44.3) [121.9 – 135.2]	
Normal (< 190)	2,518	(91.6%)	2369	(92.0%)	149	(85.1%)
Raised (190 - 240)	225	(8.2%)	201	(7.8%)	24	(13.7%)
High (> 240)	7	(0.2%)	4	(0.2%)	2	(1.1%)
**SBP range (mmHg)**						
Mean (SD) [95%CI]	124.0 (10.2) [123.6 – 124.3]		124.2 (10.1) [123.8 – 124.6]		120.3 (10.2) [123.6 – 124.3]	
Normal < 120	1,199	(43.6%)	1,200	(46.6%)	86	(49.1%)
Pre-hypertension (120-139)	1,314	(47.8%)	1,234	(48.0%)	72	(41.1%)
Stage 1 Hypertension (140-159)	226	(8.2%)	132	(5.1%)	17	(9.7%)
Stage 2 Hypertension (160-179)	9	(0.4%)	7	(0.3%)	0	(0.0%)
**DBP range (mmHg)**						
Mean (SD) [95%CI]	79.2 (8.3) [78.9 – 79.5]		79.2 (8.4) [78.9 – 79.5]		79.3 (8.7) [78.0 – 80.6]	
Normal (< 80)	1,639	(59.6%)	1,541	(59.9%)	119	(68.0%)
Pre-hypertension (80-89)	895	(32.7%)	842	(32.7%)	43	(24.6%)
Stage 1 Hypertension (90-99)	208	(7.6%)	185	(7.2%)	13	(7.4%)
Stage 2 Hypertension (100-109)	7	(0.3%)	6	(0.2%)	0	(0.0%)
**Tobacco use**						
Yes	521	(18.9%)	513	(19.9%)	8	(4.6%)
No	2,228	(81.1%)	2,061	(80.1%)	167	(95.4%)
**Number of cigarettes/ day**	**(N = 521)**		**(n = 511)**		**(n = 8)**	
< 10	266	(51.1%)	265	(51.7%)	1	(12.5%)
≥ 10	255	(48.4%)	248	(48.3%)	7	(87.5%)
**Alcohol intake**						
Yes	1,104	(40.2%)	1,052	(40.9%)	52	(29.7%)
No	1,645	(59.8%)	1,522	(59.1%)	123	(70.3%)
**Daily quantity of alcohol**	**(N = 1104)**		**(n = 1052)**		**(n = 52)**	
< 4 standard units	900	(81.5%)	862	(81.9%)	38	(73.1%)
≥ 4 standard units	204	(18.5%)	190	(18.1%)	14	(26.9%)

N/n= sample size; SD= standard deviation statistic

**Figure 2:**
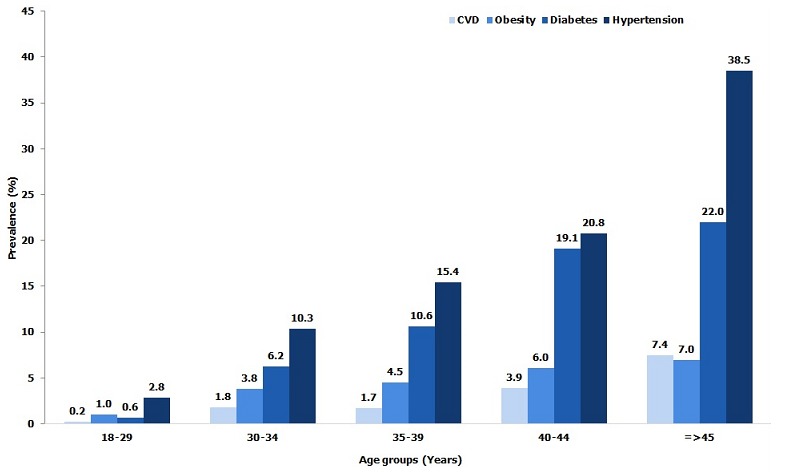
Prevalence of ODH and CVD by age groups in the TFM workforce

**Figure 3:**
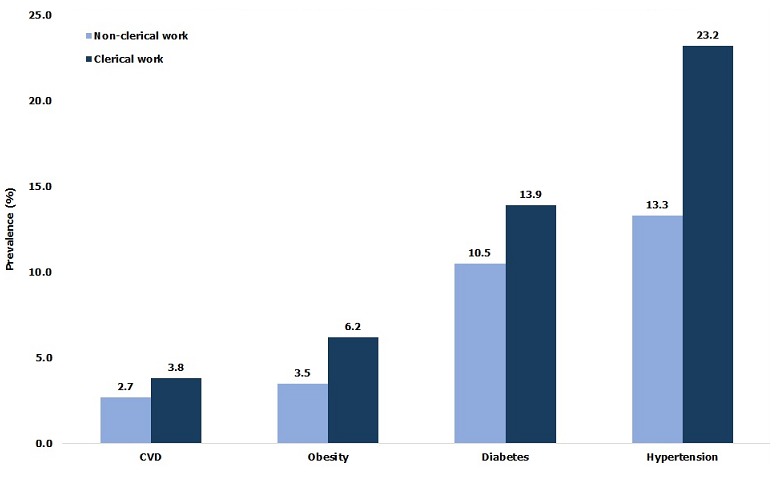
Prevalence of ODH and CVD by nature of work in the TFM workforce

**Figure 4:**
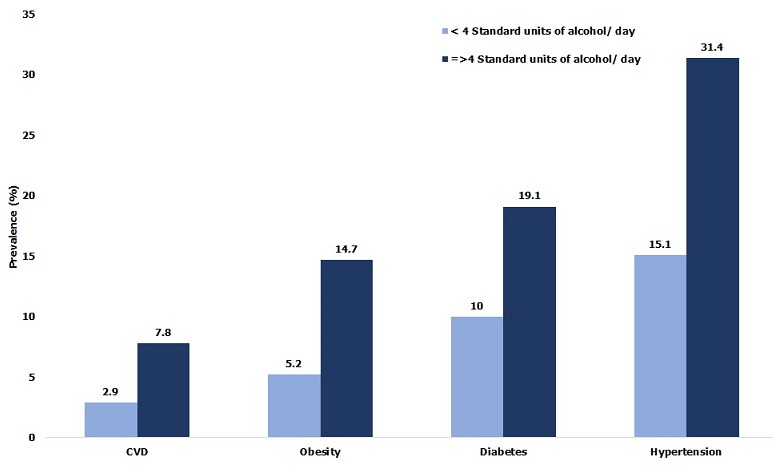
Prevalence of ODH and CVD by reported daily alcohol use in the TFM workforce

**Figure 5:**
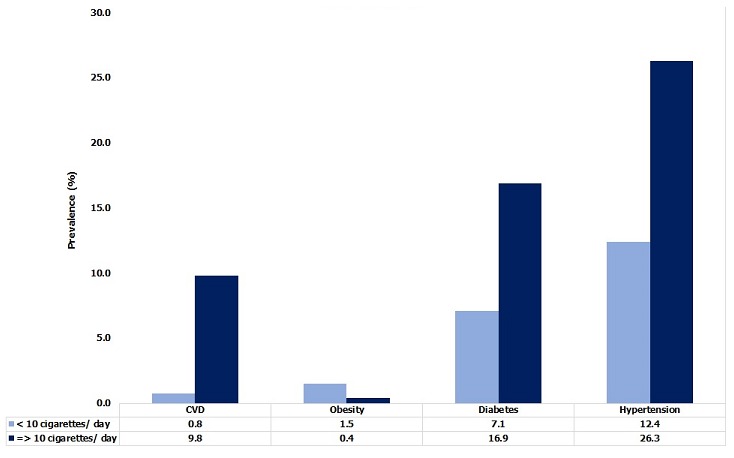
Prevalence of ODH and CVD by reported daily number of cigarettes in the TFM workforce

**Obesity, diabetes and hypertension:** Among study subjects, 19.7% of individuals were defined as overweight “pre-obese” (BMI: 25 - 29.9 kg/m^2^); 16.5% were pre-diabetic (fasting glucose: 101-125 mg /dl) and 47.8% were pre-hypertensive (SBP: 120-139 mmHg and or DBP: 80-89 mmHg) (Table 2). Table 3 presents the actual burden of obesity, diabetes, hypertension and cardiovascular disease (CVD) in the 2,749 subjects medically screened. The overall prevalence of obesity was 4.5% and significantly (p < 0.0001) greater among females (13.2%) than males (3.9%). The overall proportion of diabetics was 11.7%; again, females were significantly (p < 0.05) more affected than males (16% versus 10.2%). The overall prevalence of CVD was 3.2% with females having a significantly (p < 0.05) higher rate than males. The workforce prevalence of hypertension was 18.2% and significantly (p < 0.05) higher in males compared to females. The distribution by age found that the 40-44 and ≥ 45-year age groups had significantly (p < 0.0001) higher prevalence for ODH and CVD than ages below 40 (Figure 2). Distribution by professional categories showed that managers had greater prevalence for CVD (4.5%), hypertension (38.8%) and diabetes (17.8%) than foremen and general labor (p < 0.05), while prevalence of obesity was greater in foremen (6.9%) than in the other two categories (p < 0.05). When considering nature of work (Figure 3), clerical workers had a higher prevalence for ODH than non-clerical (p < 0.05), but no difference has been found for the prevalence of CVD between the two groups (p = 0.11). Figure 4 compares ODH and CVD in counterparts reporting drinking less than 4 standard units of alcohol per day with those consuming more. Those drinking 4 or more units of alcohol per day had a significantly (p < 0.00001) higher prevalence of obesity (14.7 % vs 5.2%), diabetes (19.1% vs 9.7%), hypertension (31.4% vs 15%) and CVD (7.8% vs 2.8%). Figure 5 shows that smokers who consumed 10 or more cigarettes per day had a significantly higher prevalence for diabetes (p < 0.005), hypertension (p < 0.0001) and CVD (p < 0.0001). However, the proportion of obese workers was significantly higher among non-smokers than smokers (4.3% vs 0.2%, p < 0.0001), whereas there was no significant difference in obesity rates among smokers and number of cigarettes used per day (p = 0.193).

**Table 3:** Prevalence of ODH and CVD in the TFM workforce (2010)

Variable	Total (N=2749)		Males (n=2574)		Females (n=175)	
Overweight (BMI ≥ 25 – 29.9 kg/m²)	541	19.7%	505	19.6%	36	20.6%
Obesity (BMI ≥ 30 kg/m²)	124	4.5%	101	3.9%	23	13.2%
Diabetes (fasting glucose ≥ 126 mg/dl or diagnosed diabetic)	322	11.7%	294	11.4%	28	16.0%
Hypertension (SBP ≥ 140 mmHg and/or DBP ≥ 90mmHg or known hypertensive taking medication)	499	18.2%	470	18.3%	29	16.6%
CVD (history of diagnosed CVD)	88	3.2%	80	3.1%	8	4.6%

N/n= sample size

## Discussion

This is the first known assessment and description of primary NCDs in a mining industry workforce in the DRC. The objective of this study was to describe the 'baseline' prevalence of obesity, diabetes, hypertension and associated risk factors in the TFM workforce from medical screening in 2010. This important data set represents “t = 0”, near the beginning of the mine production phase, a project that has an anticipated economic life running several decades or longer. This baseline information will be used for a subsequent 6-year retrospective cohort time series study based on employees' annual medical examination data archived at the TFM Occupational Health Clinic from January 2010 to December 2015. Medical records of 2,749 employees, all of whom underwent an occupational health examination between January and December 2010, were included in the study. Results show a relatively high prevalence of the three specific NCDs in this mine employee population. The overall prevalence of hypertension (18.2%) and mean values of SBP (124 mmHg) and DBP (78.7 mmHg) in this workforce were higher compared to a large mining workforce located in Papua, Indonesia [19] and communities in DRC [20, 21] and Fungurume [13]. However, a study of Congolese adults in South Kivu (eastern DRC) reported a nearly 40.1% prevalence. DRC is a geographically large and diverse country, thus regional differences in NCD prevalence would not be entirely surprising. For example, the high rate of hypertension in South Kivu may be related to altitude in eastern DRC (a mountainous region) where hypoxia and polycythaemia (chronic erythremia) can lead to vascular resistance by increase in blood viscosity [22]. In addition, the chronic stress resulting from long years of war and civil unrest in this region might be a significant contributor to hypertensive states. The proportion of diabetics in the TFM workforce was 11.7%, with mean fasting blood glucose of 93.9 mg/dl. Studies in the general population of DRC have reported a diabetes' prevalence ranging from 3.5 to 7% [21, 23, 24]. Our findings show a much higher prevalence than those reported in rural areas of sub-Saharan Africa, which are typically less than 3% [24-26]. The prevalence of diabetes and mean fasting glucose reported from TFM are also higher than those reported in mine workers in Papua, Indonesia (2.5% diabetics and mean 87.1 mg /dl fasting blood glucose) [19].

At TFM, overweight (19.7%) and obesity (4.5%) prevalence among mine workers is much higher than those reported for the general population of the DRC. For example, in the South Kivu region, obesity rates only reach 3.5% [27]. In the city of Lubumbashi (DRC), a location approximately 180km from the TFM concession, obesity (13.3% in adult females) was significantly associated with urbanization [28]. The prevalence of obesity in our study is lower than that reported in the COPPER survey conducted in Papua (6.8%) [19], but comparisons with a location and population in Indonesia (Australasia) should be viewed with caution. Unfortunately, there are few other studies on mine workforce populations from which to compare. In our study, the prevalence of smoking (18.9%) and alcohol intake (40.2%) are higher than in the general population of the DRC that shows 8% and 10% for smoking and alcohol intake, respectively [29, 30]. The TFM workforce obtains a regular income and is relatively well-paid compared to the general population. However, working and residing for long periods in a rural, isolated location, with many separated from their family during those periods, may explain in part, the relatively high rates of alcohol and tobacco use. Moreover, unrestricted access to food provided by the mine company may be responsible for a diet richer in sugar, salt and fat than usual [16, 17] thus inadvertently promoting increases in overweight and obesity rates in the workforce. As with other studies, we confirm the role of age, professional grade, nature of work (clerical/ non-clerical), smoking and alcohol use as major risk factors for NCDs [31-37]. However, obesity decreased with an increase in the amount of tobacco used. The lower probability of weight gain in smokers does not compensate for the grave health consequences of tobacco use. This study demonstrates the relatively high prevalence of certain risk factors for NCDs in a mining workforce in DRC, as smoking and alcohol abuse increase the risk of developing CVD and other NCDs [30, 38]. Our results also indicate that public health efforts are needed to identify appropriate measures to reduce tobacco use and excessive alcohol intake, such as public awareness campaigns highlighting the harmful effects of both and methods to reduce or eliminate use. Primary limitations of this study were: working with archived retrospective data that had not been collected by study team, therefore some systematic errors cannot be excluded. Quantitative medical examination data were collected and entered in standardized fashion by qualified health professionals attached to the Occupational Health Clinic (OHC). For subjective entries, some different biases (recall, interviewer, measurement, etc). Moreover, questions regarding smoking and alcohol use are prone to study subject reporting bias.

Additionally, the study only included employees who had a completed medical examination record in 2010, either for purposes of initial employment recruitment or post-employment follow-up at the OHC. How many in the workforce might have been excluded (and for what reasons) is not known. Data on occupational exposure were available for 606 (22%) study subjects; however, it is unclear if this is truly reflective of the overall workforce exposure profile. Lastly, due to the composition of the workforce, only 6.4% (n = 175) of study subjects were females, therefore under-representative compared to the general population, thereby making extrapolations and comparisons with male counterparts problematic. The promotion of healthful lifestyles through various forms of media and awareness education could help reduce the prevalence of overweight and obese employees and in the general population. In recent decades, traditional societies in many developing countries have experienced rapid and poorly planned urbanization, leading to lifestyles characterized by poorer nutrition, reduced physical activity, increased stress, pollution, and increased consumption of tobacco and alcohol. These key behavioral risk factors contribute to the increased occurrence of some of the leading NCDs. To combat this, some countries have introduced taxes on unhealthy foods and beverages in attempts to reduce their consumption [39]. In a more controlled work environment, like a mine, a variety of measures can be taken to decrease the burden of preventable NCDs such as establishing and enforcing workplace policies on use and controlled access to tobacco and alcohol. Food quantity (portions) and preparation can also be controlled (e.g. reduction in high fat, salt and sugar content) and monitored in a workforce reliant on primary nutritional provision from a company-sponsored provider. Lastly, promotion of various activities, such as regular physical exercise, can be organized at the company and departmental levels. In other words, a variety of activities and initiatives are possible in a workplace that might not be possible in the general population. Organized workplace programs that integrate health risk assessment measures with specific interventions (e.g. targeting dietary intake and increasing physical activity) are effective means to help abate the looming crisis of NCDs, with concomitant improvements in overall medical parameters promoting psychological and physical wellbeing [40]. For the employer, moreover, programs that improve health outcomes can lead to reductions in absenteeism and associated health care costs, while increasing productivity.

## Conclusion

This baseline study found pronounced high rates of obesity, diabetes mellitus, hypertension (ODH) and associated risk factors in a mining workforce compared to the general population of DRC. This study also provides evidence of the relationship between NCDs and various socio-demographic, occupational, anthropometric, biomedical and behavioral risk factors, in the target population. Given the conspicuous high rate of ODH and linked risk factors, the implementation of a comprehensive NCD response program is crucial to abate this increasing health burden. Programs promoting lower alcohol use, smoking cessation, reduced salt, sugar and fat intake, together with periodic blood pressure, cholesterol and weight monitoring should be implemented. Further research is needed to monitor the trends and burden of NCDs and their risk factors in this population of mine workers.

### What is known about this topic

- Obesity, diabetes mellitus and hypertension are major risk factors for cardiovascular diseases worldwide resulting in an estimated 18 million deaths annually;
- Mining is a relatively hazardous activity, both in terms of potential injuries and deaths, but also increased risk for developing NCDs;
- Various risk factors (environmental, lifestyle, etc) associated with mining have been implicated in the increased occurrence of NCDs.

### What this study adds

- This is the first known study that describes the prevalence of obesity, diabetes, hypertension, all chronic conditions associated with increased risk for cardiovascular disease and contributing risk factors in a mining workforce population in the Democratic Republic of Congo;
- Rates of obesity, diabetes mellitus, hypertension and associated risk factors in the TFM workforce are high compared to the general populations, but varied significantly by age;
- Given the high proportion of employees with excess weight, pre-hypertensive and pre-diabetic conditions, a shift to a higher prevalence of patent obesity, diabetes and hypertension is anticipated to occur without implementation of a mitigation program to reduce known risk factors.

## Competing interests

The authors declare no competing interests.
